# Initial periodontal screening and radiographic findings - A comparison of two methods to evaluate the periodontal situation

**DOI:** 10.1186/1472-6831-11-3

**Published:** 2011-01-14

**Authors:** Dirk Ziebolz, Ivette Szabadi, Sven Rinke, Else Hornecker, Rainer F Mausberg

**Affiliations:** 1 Department of Preventive Dentistry, Periodontology and Cariology, University Medical Centre Goettingen, Germany; 2 Private Dental Practice, Hanau, Germany

## Abstract

**Background:**

The periodontal screening index (PSI) is an element of the initial dental examination. The PSI provides information on the periodontal situation and allows a first estimation of the treatment required. The dental panoramic tomography (DPT) indicates the proximal bone loss, thus also allowing conclusions on the periodontal situation. In this study, the results of both methods in determining the periodontal situation are compared.

**Methods:**

The clinical examination covered DMF-T, QHI, and PSI scores at four proximal sites per tooth; the examining dentist was unaware of the radiographic finding. Based on the PSI scores, the findings were diagnosed as follows: score 0 - 2 "no periodontitis", score 3 and 4 "periodontitis". Independent of the locality and time of the clinical evaluation, two dentists examined the DPTs of the subjects. The results were classified as follows: no bone loss = "no periodontitis", and bone loss = "periodontitis".

**Results:**

112 male subjects (age 18 to 58, Ø 37.7 ± 8 years) were examined. Regarding the PSI, 17 subjects were diagnosed "no periodontitis" and 95 subjects "periodontitis". According to the evaluation of the DPTs, 70 subjects were diagnosed "no periodontitis" and 42 "periodontitis". A comparison of both methods revealed that the diagnosis "no periodontitis" corresponded in 17 cases and "periodontitis" in 42 cases (53%). In 47% (53 cases) the results were not congruent. The difference between both methods was statistically significant (p < 0.001; kappa = 0.194).

**Conclusion:**

The present study shows that the initial assessment of the periodontal situation significantly depends on the method of evaluation.

## Background

The patient's quality of life is markedly influenced by tooth loss. Reduced chewing ability, inferior aesthetics and the need for a prosthetic restoration place a burden on those affected [1]. Tooth decay and periodontitis are the main reasons for tooth loss. Throughout the world, caries and periodontal diseases are the most common diseases of all [2,3]. Because of preventive measures, it has been possible to achieve a noticeable decrease in caries, but the level of periodontal diseases still remains extremely high.[4] Therefore, early diagnosis is essential for the therapy of periodontitis, in order to avoid a high incidence and progression of the disease [5-8]. However, even today, periodontitis is often diagnosed quite late. In many cases, periodontal problems become apparent for the first time when symptoms of severe periodontitis appear, such as, for example, increased tooth mobility or tooth migration. Therefore, the identification of periodontitis at an early stage is essential to halt the further progress of the disease. However, no gold standard has been established or even defined up until now. Research evidence shows that screening with panoramic radiography is unproductive, with a majority of patients receiving no diagnostic benefit or treatment impact.[9,10]. As the initial bone loss is often underestimated, dental panoramic tomography (DPT) has less value for the diagnosis of initial periodontal lesions [11-14]. However, DPT is a method of demonstrating past disease activity in form of bone loss [15]. In this cases, panoramic radiography may be helpful for the diagnosis, characterization and monitoring of advanced periodontitis [11,12,16,17].

In 1992, the American Dental Association (ADA) and the American Academy of Periodontolgy (AAP) announced the "Periodontal Screening & Recording - PSR^®^" ("Periodontal Screening Index (PSI)" in Germany) - a modification of the CPITN (Community Periodontal Index of Treatment Needs) - as a "simple, effective system to detect periodontal disease" [18-20]. Periodontal screening (PSR^®^/PSI) provides detailed information about the condition of a patient's periodontium and allows a quick and comprehensive evaluation of the periodontal situation [18-20]. With the PSR^®^/PSI, even the earliest symptoms of periodontal disease can be detected clinically. Moreover, it allows a first estimate of the periodontal treatment required [18-20].

In the present study, the reliability of the PSR^®^/PSI in early diagnostics is compared with conventional radiographic diagnostics using DPTs. The comparison is made in relation to the diagnoses "periodontitis" and "no periodontitis". The reasons for possibly aberrant diagnoses are discussed.

## Methods

This is a retrospective clinical trial comparing two diagnostic methods to evaluate the periodontal situation. The study was performed at a dental office of the "Bundeswehr"/German Federal Armed Forces (Munster, Lower Saxony, Germany). For all professional soldiers the DPT are elements of the basic dental examination.

### Participants

The dental panoramic tomographies (DPTs) of all subjects to be included in the study were required to have taken place no longer than 12 months prior to the clinical examination of this study. X-rays with procedural errors or with an indistinct anterior region were rejected. Subjects with removable dental restorations and those who had already undergone a periodontal therapy or dental prophylaxis appointments were also excluded. After informed consent, 112 professional soldiers (aged 18 to 58 years, mean: 37.7 years) were recruited according the above criteria. The ethics committee of the Georg-August-University Goettingen, Germany, approved the study (application No. 11/9/04).

### Clinical examination

All subjects were examined once under standardized conditions by a dentist (IS), who was calibrated prior to the examination (kappa value > 0.8). The smoking habits of those included in the study were assessed. DMF-T, periodontal screening PSR^®^/PSI and the modified Quigley-Hein-Index according to Turesky et al. [21,22] (QHI) were recorded.

The PSR^®^/PSI was taken with the WHO probe (Morita, Kyoto/Japan) [18-20]. Every tooth was probed at four sites (mesio-vestibular, disto-vestibular, mesio-oral and disto-oral) and the PSR^®^/PSI score (0 to 4) was recorded. The highest score was determined for each sextant. PSR^®^/PSI scores 3 and 4 distally of the second molars were not taken into consideration, in order to avoid a false-positive finding due to "pseudo pockets", which often occur in this region. According to the definition of Cutress et al., [23] which states that the highest PSR^®^/PSI score of a subject should determine the clinical diagnosis, the following classifications were made for each participant in the study: PSR^®^/PSI score 0, 1, and 2: "no periodontitis"; PSR^®^/PSI score 3 and 4: "periodontitis". At the time of the clinical evaluation, the radiographic findings for the subjects were not known to the dentist.

### Radiographic examination

The evaluation of DPTs in relation to bone loss was carried out under standardized conditions, in a shaded room with an x-ray film viewer capable of functioning under such conditions. The DPTs were examined by two dentists (DZ and RM) by dual (consensus) reading. Both examiners worked "blind", i.e. they were not informed about the result of the clinical examination. All x-rays were examined twice at a 14-day interval. As is the case in routine daily practice, the level of bone loss was evaluated subjectively. For each tooth, the distance between the alveolar crest (AC) and the cemento-enamel junction (CEJ) was examined mesially as well as distally. The dentists evaluated whether the participant suffered from periodontal bone loss or whether the findings were due to normal anatomy. Based on the x-ray evaluation, a radiological diagnosis followed the clinical examination using the classification "no bone loss - no periodontitis" or "bone loss - periodontitis". The DPTs were evaluated according to the recommendation of Pepelassi and Diamanti-Kipioti: [24] "no bone loss - no periodontitis": bone level proximally at the physiological height (distance CEJ-AC up to 3 mm) and "bone loss - periodontitis": bone level proximally reduced (distance CEJ-AC > 3 mm).

### Statistical evaluation

Statistical analysis was performed with the commercially available program SPSS 14.0 (SPSS, Inc., Chicago, IL, USA). The statistical comparison of the clinical and the radiographic diagnoses was made using three test procedures. The two methods of evaluation were compared with the McNemar test and p < 0.05 was defined as statistically significant. The kappa value was determined as the degree of congruence of the two methods of evaluation; value of > 0.80 was considered to indicate very good congruence. In addition, the Kruskal-Wallis test was applied for the calculation and determination of possible interactions, i.e. DMF-T and oral hygiene as well as the examined region (p < 0.05).

## Results

### Clinical examination

DMF-T and oral hygiene: The mean DMF-T of the participants was 11.8 ± 4.8 (DT = 0.5 ± 0.4, MT = 1.3 ± 1.9, FT = 10.7 ± 4.9). The mean QHI was 2.3 ± 0.9 (min: 1; max: 5). Periodontal screening (PSR^®^/PSI): No subject was considered to be healthy in terms of periodontics (PSR^®^/PSI score 0). Three subjects (2.7%) had the highest PSR^®^/PSI score of 1.14 subjects (12.5%) had a PSR^®^/PSI score of 2. 72 subjects (64.3%) had a PSR^®^/PSI score of 3, while 23 subjects (20.5%) had a PSR^®^/PSI score of 4. According to the definition used, 17 subjects (15.2%) were diagnosed as having "no periodontitis" and 95 subjects (84.8%) were diagnosed as having "periodontitis".

### Radiographic examination

In 70 x-rays, representing 62.5% of cases, no bone loss was established, which resulted in the classification: "no periodontitis". In 42 x-rays (37.5%), bone loss was observed and was classified as "periodontitis".

### Comparison of clinical and radiographic findings for all subjects

The clinical diagnoses and the diagnoses made using DPTs were compared according to the diagnoses "no periodontitis" and "periodontitis". The McNemar test revealed that both methods differed significantly (p = 0.00). The kappa value (kappa = 0.194) also revealed only a low congruence. In 17 subjects (15.2%), the clinical examination as well as the radiographic findings produced the diagnosis "no periodontitis" (Table 1). Therefore, this group was referred to as congruence "no periodontitis". In 42 subjects (37.5%) the clinical examiner, as well as the two radiographic examiners, diagnosed "periodontitis" (Table 1). This group was accordingly referred to as congruence "periodontitis". No congruence was established for the remaining 53 subjects (47.3%) (Table 1): the clinical diagnosis was "no periodontitis" while the radiographic finding was "periodontitis". The combination "clinical finding - periodontitis"; "radiographic finding - no periodontitis" did not arise.

**Table 1:** Assigning the diagnoses "no periodontitis" and "periodontitis" to the subjects (n = 112) based on the two methods of examination (PSR^®^/PSI and DPT)

**PSR**^®^**/PSI DPT**	**"no periodontitis"**	**"periodontitis"**
**"no periodontitis"**	**n = 17 (15.2%)**	n = 53 (47.3%)
**"periodontitis"**	n = 0	**n = 42 (37.5%)**

### Comparison of clinical and radiography findings according to DMF-T, oral hygiene and to the region examined

**Figure 1:**
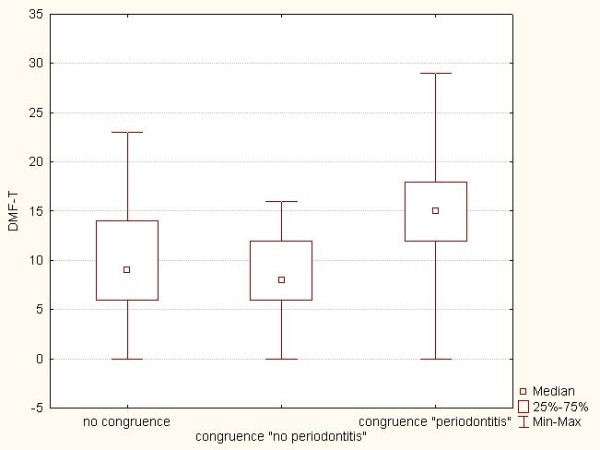
Box plots showing the DMF-T of subjects in the three groups: *congruence *"no periodontitis", *congruence *"periodontitis", and "no congruence"

DMF-T: The mean DMF-T of the group congruence "no periodontitis" was 9.1 ± 4.3 (DT = 0.1, MT = 0.4, FT = 8.6) and that of the group "no congruence" 9.9 ± 4.9 (DT = 0.5, MT = 0.5, FT = 8.9). The mean DMF-T of the group congruence "periodontitis" was 16.1 ± 5.1 (DT = 0.2, MT = 1.8, FT = 14.1) (Table 2), and so was significantly higher than that of the other two groups (p < 0.001). No significant difference was found between the groups congruence "no periodontitis" and "no congruence" (p = 1.0) (Figure 1).

**Table 2:** Age, DMF-T and QHI of the subjects in the three groups: *congruence *"no periodontitis", *congruence *"periodontitis", and "no congruence"

**Group**	**Age [years] **(mean ± sd)	**DMF-T **(mean ± sd)	**QHI **(mean ± sd)
***congruence*"no periodontitis" (n = 17)**	24 ± 6	9.1 ± 4.3	1.4 ± 0.5
***congruence *"periodontitis" (n = 42)**	42 ± 8.5	16.1 ± 5.1	2.7 ± 1.4
**"no congruence" (n = 53)**	28 ± 8	9.9 ± 4.9	2.3 ± 0.9

#### Oral hygiene

**Figure 2:**
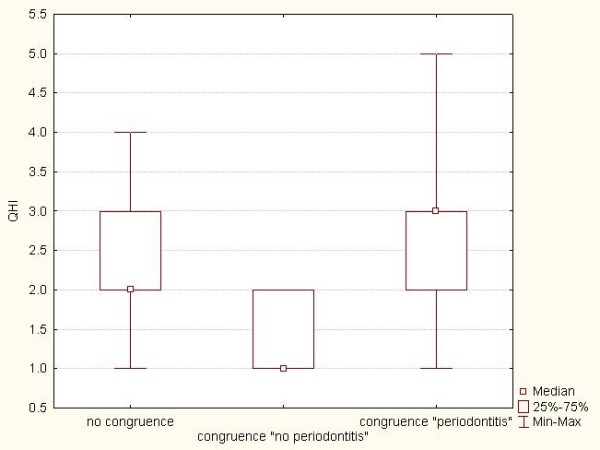
Box plots showing the QHI of subjects in the three groups: *congruence *"no periodontitis", *congruence *"periodontitis", and "no congruence"

The mean QHI of the group congruence "no periodontitis" was 1.4 ± 0.5 (min: 0; max: 2). The group "no congruence" had a mean QHI of 2.3 ± 0.9 (min: 1; max: 4), while the mean QIH of the group congruence "periodontitis" was 2.7 ± 1.4 (min: 1; max: 5) (Table 2). The level of oral hygiene of the group congruence "no periodontitis" was significantly better than that of the two other groups (p < 0.001). The groups "no congruence" and congruence "periodontitis" did not show a significant difference (p = 0.079) (Figure 2).

#### Examined region

The highest PSR^®^/PSI score for the group congruence "no periodontitis" was 2. Four percent of the sites showed healthy conditions (PSR^®^/PSI score 0), the majority (79.3%) having a score of 1 (bleeding on probing, indicating gingivitis). 16.7% of the sites had a PSR^®^/PSI score of 2. In the group "no congruence", a PSR^®^/PSI score of 3 was found at 59.2% of the sites. In the anterior sextants, only a PSR^®^/PSI score of 0 to 2 was found. A PSR^®^/PSI score of 4 was only found at 2% of the sites. In the group congruence "periodontitis ",PSR^®^/PSI scores of 3 and 4 were also found in the anterior sextants. In the posterior sextants, a PSR^®^/PSI score of 3 was found in 61.7%, score 4 was found at 11.5% of the sites.

## Discussion

According to the PSI evaluation, 17 subjects (15.2%) were diagnosed as having "no periodontitis" while 95 subjects (84.8%) were found to have "periodontitis". The high percentage of subjects with "periodontitis" seems to be due to the strict diagnosis. In terms of initial diagnostics, a strict attribution of "no periodontitis" for the PSR^®^/PSI scores 0, 1 and 2 and "periodontitis" for PSR^®^/PSI scores of 3 and 4 seems reasonable. However, in relation to the extent of treatment, there is a considerable difference depending on whether one or more sites of a tooth are assigned a score of 3 or 4.

In 70 (62.5%) of the 112 subjects, the radiographic diagnosis established "no periodontitis", and 42 subjects (37.5%) were diagnosed as having "periodontitis". Just as in daily practice, in the present study, the radiographic findings of the DPTs were made by measuring the distance between the cemento-enamel junction (CEJ) and the alveolar crest (AC). As the assessment of DPTs by different examiners could result in significant deviations, this evaluation was performed by two dentists using identical criteria for assessment, in order to achieve a high degree of objectivity [14].

The results demonstrate that both methods differ significantly from one another. In 59 subjects (52.7%), congruence was found in relation to the diagnoses "no periodontitis" and "periodontitis", while in 53 subjects (47.3%) this was not the case. Accordingly, three groups were obtained: congruence "no periodontitis", congruence "periodontitis", and "no congruence". In contrast to Walsh et al. [25] , who performed a similar study, the combination clinical diagnosis: "no periodontitis" and radiographic diagnosis: "periodontitis" was not found in the present study. Walsh et al. [25] examined the correlation between bone loss on the DPT and the clinical finding using CPITN. The results revealed that bone loss on the DPT was closely related to the CPITN scores [25]. However, the calculated loss of bone structure was higher on the DPT than with the corresponding CPITN score. The authors therefore recommended the use of DPT for periodontal diagnosis [25]. The results of Walsh et al. [25] were confirmed by our study only in relation to the group congruence "no periodontitis". In this group, the highest PSR^®^/PSI score was 2. In the study of Walsh et al. [25] only posterior sextants were examined and diagnosed, respectively. Moreover, the assessment of the distance between the cemento-enamel junction (CEJ) and the alveolar crest (AC) was evaluated with special reference splints. Afterwards, the distance CEJ-CA was statistically allocated in correlation to the root length and the magnification factor of the DPT [14]. The results showed that the bone loss on the DPT was closely related to the CPITN scores [25]. However, the calculated loss of bone structure was higher on the DPT than the respective CPITN score. This led to the conclusion that referring to the CPITN scores 0, 1, and 2, no difference in the distance CEJ-CA exists and thus no bone loss can be detected in the x-ray. The authors therefore recommend the DPT for periodontal diagnosis [25]. However, accommodating daily routine in our study the radiographic finding was performed only by evaluating the distance between the cemento-enamel junction and the alveolar crest without any aids. This subjective assessment might be considered a weak point in diagnosis based on the radiographic finding. Moreover, it must be considered that x-rays only provide information on osseous structures/bone loss while the PSI reflects the current clinical situation [11,25]. According to Lange, [12] x-rays are only of limited value in the detection of early periodontal bone loss. In DPTs, an initial loss of proximal bone is often either not detected or is underestimated, and even moderate lesions in the facial and/or oral direction are often not identified [11,13,14,26]. However, in patients with advanced bone loss, the DPT yields reliable results [11,16,17].

All 42 subjects with the radiographic diagnosis "periodontitis" were identified as having periodontal disease using the PSR^®^/PSI, as well (congruence "periodontitis"). In this group, the majority of subjects had PSR^®^/PSI scores of 3 or 4, mainly in the posterior sextants. This result supports the findings of studies indicating that progressive bone loss can be reliably diagnosed using DPT [11,16,17]. In the "no congruence" group, all subjects were clinically diagnosed as having "periodontitis" whereas the radiographic evaluation revealed "no periodontitis". Walsh et al. [25] reported similar findings. In this group, a PSR^®^/PSI score of 3, occasionally a score of 4, was found exclusively in the posterior sextants. In the anterior region of the lower jaw, initial signs of inflammation, i.e. gingival bleeding on probing, calculus and gingival swelling with pseudo-pockets, were mostly found. These symptoms are signs of poor oral hygiene: the group "no congruence" had a significantly lower level of oral hygiene compared to the group congruence "no periodontitis". The discrepancies between the two methods of examination derive from different approaches. The PSR^®^/PSI differentiates between gingival inflammation and periodontal destruction. Therefore, the PSR^®^/PSI indicates even early symptoms of periodontal disease. According to the PSR^®^/PSI only a few participants were diagnosed having "no periodontitis". Since gingival inflammation is often accompanied with gingival swelling, i.e. pseudo-pockets; these findings PSR^®^/PSI (score 3 and 4) may only pretend "periodontitis". In this case gingivitis therapy, i.e prophylaxsis appointments (professional tooth cleaning) simply can reduce the PSR^®^/PSI scores.

According to Goodson et al., [27] the PSR^®^/PSI shows the clinical process of initial periodontal disease that will sometime later result in bone loss which can be detected radiographically. Our results are in accordance with Khocht et al.; [28] they also compared the periodontal situation with radiographs (DPT) and PSR^®^/PSI and found no correlation between the two methods. This indicates that radiographs (DPT) taken in daily dental practice are not highly reflective of the real periodontal situation [28]. In contrast to this, the PSR^®^/PSI seem to be a useful screening tool that will enhance identification of patients even with initial periodontal disease [29]. For a specified diagnosis, the characterization, the treatment and the control of advanced periodontitis, x-rays in combination with detailed clinical records are essential.

Limitation of the study: It has to be considered that x-rays cannot diagnose "periodontitis" or periodontal disease. All a radiograph can do is demonstrating the consequences of periodontitis, i.e. bone loss, and will not provide information about disease activity. This point should be taken into consideration regarding our definition of the radiographic diagnoses: "no periodontitis" and "periodontitis", respectively.

The possible gap between radiography and clinical examination (at most 12 month) may be a weakness of the study, this concerned overall only three participants. However, it is rather unlikely that radiographic features may have changed in the mean time. A change could only be related to the clinical periodontal situation.

## Conclusions

The PSR^®^/PSI is essential for initial periodontal examination. The DPT is of no value in cases of initial screening for periodontal problems. If signs of pathological changes in the periodontium are established, a radiographic examination and detailed findings are essential for further diagnostics.

## Competing interests

The authors declare that they have no competing interests.

## Authors' contributions

DZ has made substantial contributions to conception and design of the study, was one of radiographic examiners and wrote the manuscript. IS carried out the clinical examination and performed the statistical analysis. SR hasbeen involved in revising it critically for important intellectual content and have given final approval of the version to be published. EH conceived of the study, and participated in its design and coordination, interpretation of data and has been involved in drafting the manuscript. RM was the head of the study; have made substantial contributions to conception and design of the study and was one of the radiographic examiners.

All authors read and approved the final manuscript.

## Pre-publication history

The pre-publication history for this paper can be accessed here:

http://www.biomedcentral.com/1472-6831/11/3/prepub
